# Supplementary material for: Porous hypercrosslinked polymer-TiO2-graphene composite photocatalysts for visible-light-driven CO2 conversion
Source: Nat Commun. 2019 Feb 8;10:676. doi: 10.1038/s41467-019-08651-x (PMC6368626; doi:10.1038/s41467-019-08651-x)
Supplement: Supplementary file 1 — Supporting Information [file 41467_2019_8651_MOESM1_ESM.pdf]

Supplementary Information

Porous hypercrosslinked polymer-TiO_2-graphene composite photocatalysts for visible-light-driven CO_2 conversion

Wang et al.

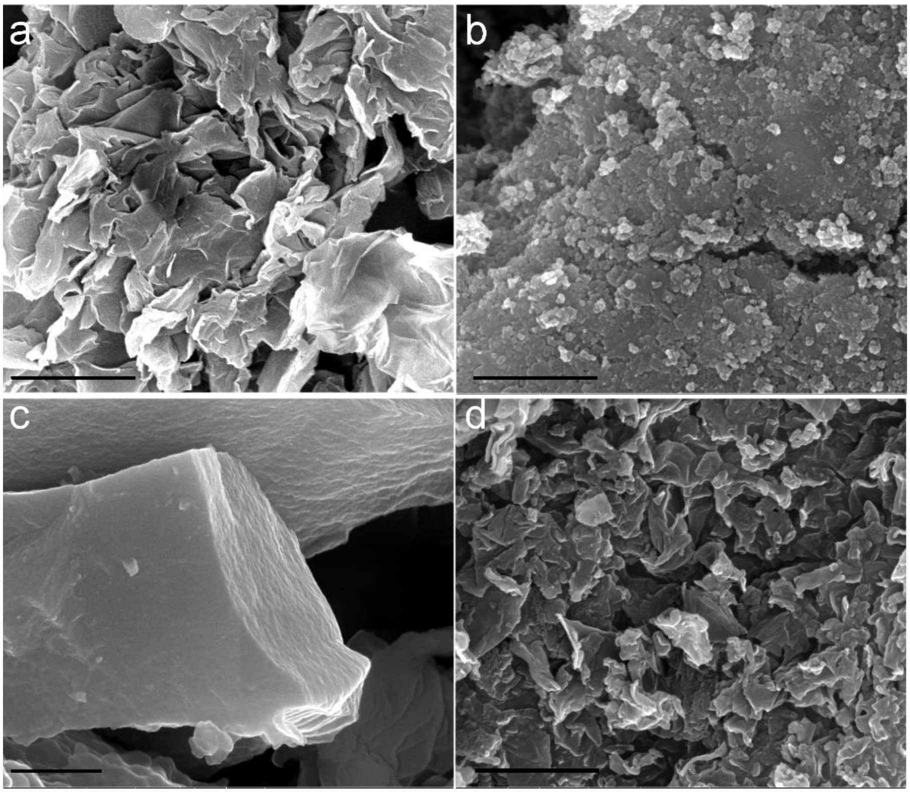

Supplementary Figure 1 SEM images of materials. SEM images of **a** GO, **b** TiO_2-G, **c** pure HCP polymer (used *syn*-$PhPh_3$ as monomer), and **d** HCP-TiO_2-FG. The scale bar are 1 μm in **a**, 1 μm in **b**, 500 nm in **c**, and 1 μm in **d**.

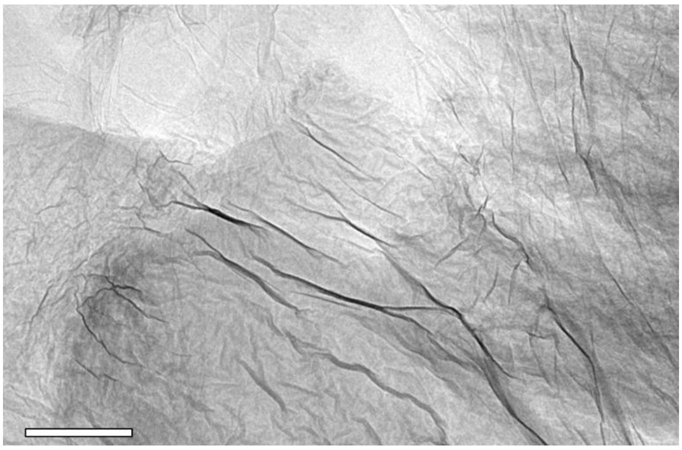

Supplementary Figure 2 TEM image of graphene nanosheets. The scale bar is 1 μm.

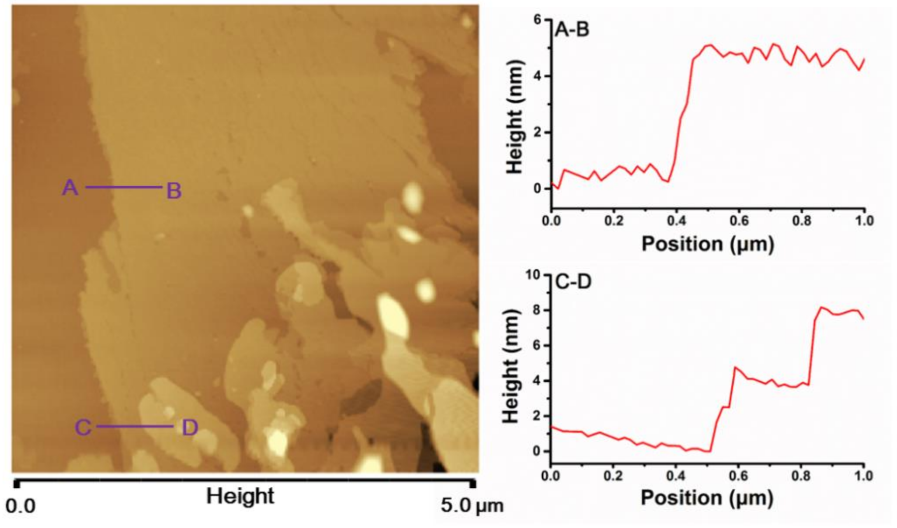

Supplementary Figure 3 The AFM images and height analysis of TiO_2-FG on mica wafer.

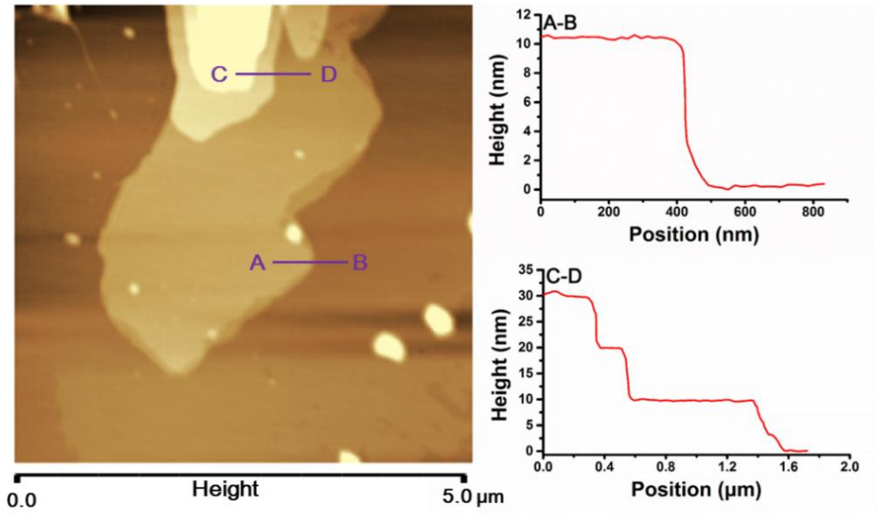

Supplementary Figure 4 The AFM images and height analysis of HCP-TiO_2-FG on mica wafer.

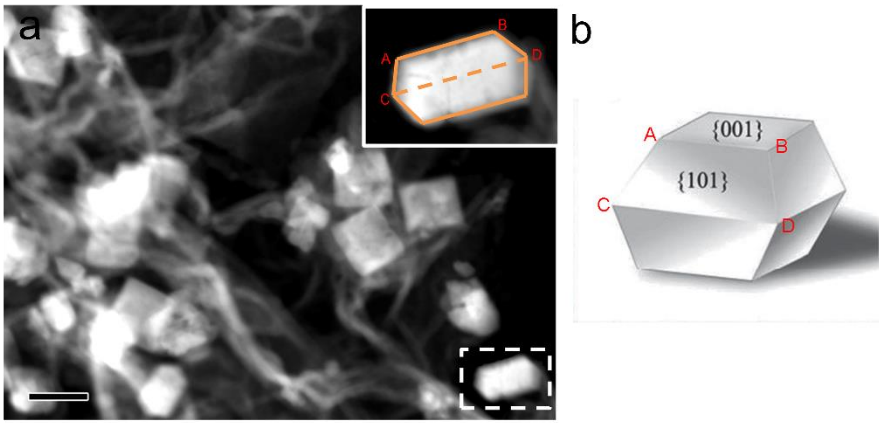

Supplementary Figure 5 a Scanning transmission electron microscopy (STEM) images of HCP-TiO_2-FG. **b** Simulated geometric model of an anatase TiO_2 crystal with co-exposed {001} and {101} facets. The scale bar is 100 nm in **a**

The percentage of the exposed {001} facets in the TiO_2 crystal was calculated using the following equation according to its simulated geometric structure[1].

$$\text{Equations1: } S_{001\%} = \frac{\cos\theta}{\csc\theta + \left(\frac{AB}{CD}\right)^{-2} - 1} = 30\ \%$$

θ is the theoretical value for the angle between the {001} and {101} facets of anatase (θ=68.2°), AB/CD is the degree of truncation.

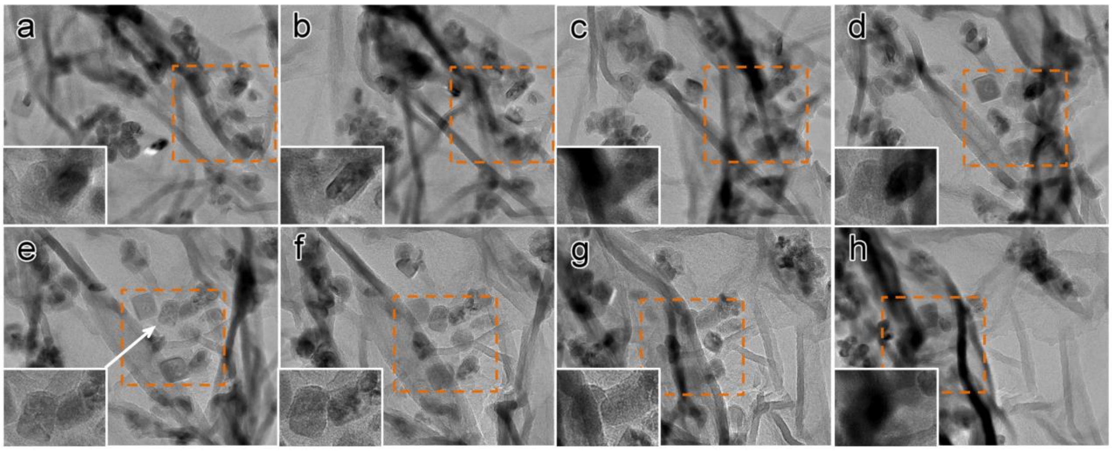

Supplementary Figure 6 Multiple views of the HCP-TiO_2-FG sample at the selected angles. -50° of **a**, -38° of **b**, -24° of **c**, -10° of **d**, 0° of **e**, 10° of **f**, 24° of **g** and 38° of **h**. The insets are the rotation of the selected two crystals that labeled by the white arrow in **e**.

By rotating the angle of the sample, multiple images were obtained to create a 3D-TEM video (Supplementary video) and a tilt series of images at the selected angles are displayed in **Supplementary Figure 6** . The TiO_2 crystals in the labeled area can be distinctly observed at the angles of around 0° (d-f). By rotating the angle far from 0°, they were gradually hidden inside the HCP layers, which could be identified by the wrinkles of the HCP outer layer (a-c and g-h).

Therefore, the TiO_2 crystals on the graphene sheets were not exposed outside but encapsulated by the ultrathin HCPs layer.

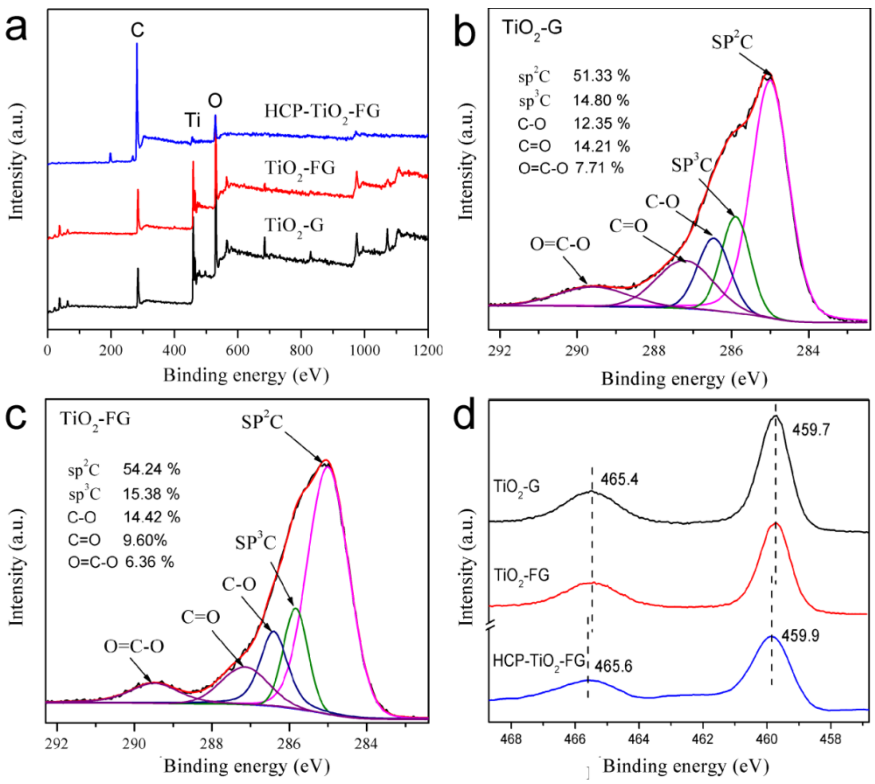

Supplementary Figure 7 XPS spectra of materials. a XPS spectra of TiO_2-G, TiO_2-FG, and HCP-TiO_2-FG**.** C^{1s} XPS spectra of **b** TiO_2-G and **c** TiO_2-FG. **d** Ti^{2p} XPS spectra of TiO_2-G, TiO_2-FG, and HCP-TiO_2-FG**.**

After curve fitting, the high-resolution C^{1s} spectrum was divided into 5 peaks. The different peaks centered at 285.0, 285.8, 286.4, 287.1 and 289.5 eV were assigned to the sp^2 C, sp^3 C and the residual oxygen-containing groups (C-O, C=O, and O=C-O) on graphene surface, respectively. The functionalization of TiO_2-G caused the ratio of sp^2 C and sp^3 C signals to be increased due to the introduction of phenyl groups on graphene. During the co-polymerization of TiO_2-FG and *syn*-$PhPh_3$, the introduction of more sp^2 carbon from *syn*-$PhPh_3$ than the methylene linker endowed HCP-TiO_2-FG much higher ratio of sp^2 C to sp^3 C signals. Meanwhile, the Ti^{2p} spectrum in the TiO_2-G sample showed two distinct peaks at 459.7 and 465.4 eV with a spin-orbital doublet splitting of 5.7 eV, corresponding to the oxidation state of +4 in TiO_2.

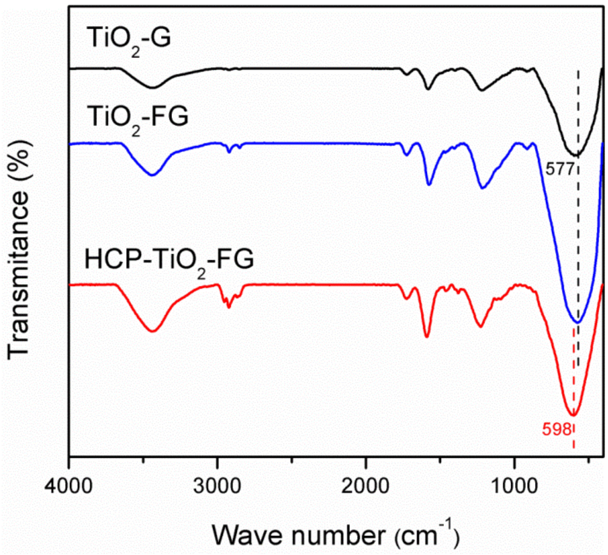

Supplementary Figure 8 FT-IR spectra of TiO_2-G, TiO_2-FG and HCP-TiO_2-FG.

The strong absorption bands at 577 cm^{-1} originate from the Ti-O-Ti stretching vibrations in these TiO_2-based composites. The characteristic peak at 1573 cm^{-1} corresponds to a C=C skeletal vibration of the graphene sheets for TiO_2-G, TiO_2-FG and HCP-TiO_2-FG. Compared with TiO_2-G, no obvious aromatic ring skeleton vibration peaks near 1485 cm^{-1} was shown for TiO_2-FG, which might be attributed to the low content of phenyl groups by functionalization. Following the copolymerization of TiO_2-FG and *syn*-$PhPh_3$, the formation of methylene and the introduction of aromatic ring skeleton of *syn*-$PhPh_3$ make HCP-TiO_2-FG show very strong C-H stretching vibrations of methylene near 2920 cm^{-1}, and aromatic ring skeleton vibration peaks near 1485 cm^{-1}.

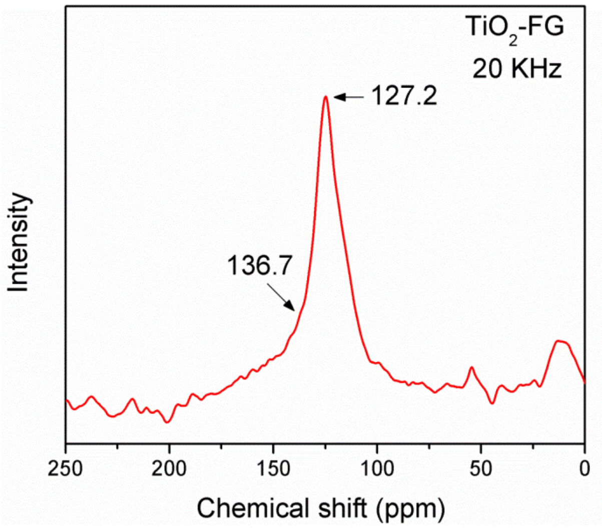

Supplementary Figure 9 ^{13}C CP/MAS NMR spectrum of TiO_2-FG.

The low graphene content in TiO_2-G and hydrogen content for graphene make the collection of ^{13}C CP/MAS NMR spectrum for TiO_2-G unsuccessful. Compared with the reported data, the

appearance of shoulder peak at 136.7 ppm can be ascribed to the introduction of phenyl groups[2]. The formation of methylene and the introduction of abundant sp^2 carbon from *syn*-$PhPh_3$ make HCP-TiO_2-FG show much stronger resonance peaks near 136.7 ppm and the appearance of peaks near 32.9 ppm.

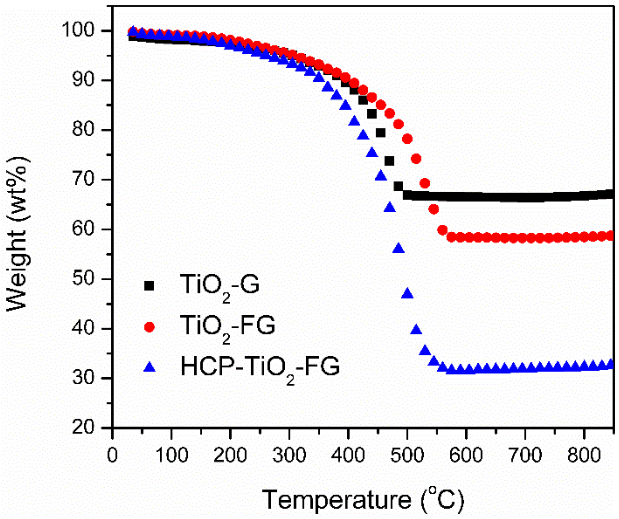

Supplementary Figure 10 Thermogravimetric analysis of TiO_2-G, TiO_2-FG, and HCP-TiO_2-FG at heating rate of 10 °C /min under air.

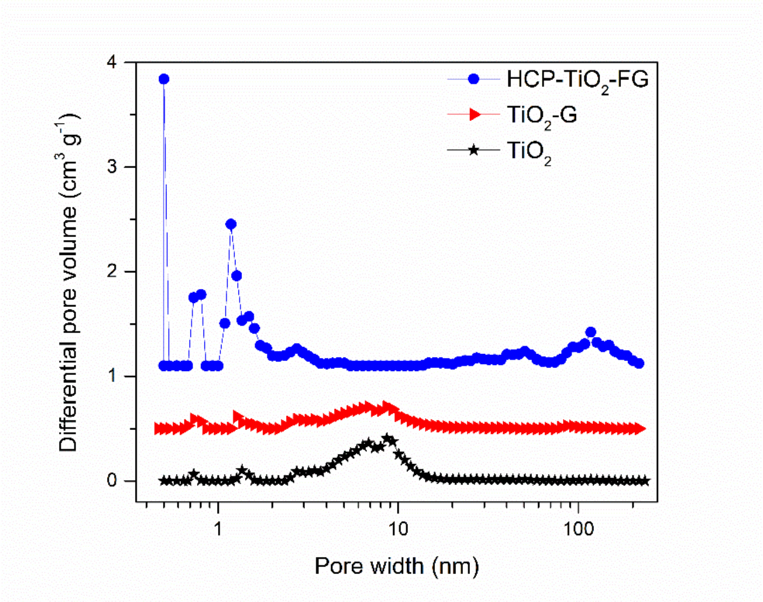

Supplementary Figure 11 Pore size distributions of TiO_2, TiO_2-G, and HCP-TiO_2-FG that calculated using DFT methods (slit pore models, differential pore volumes).

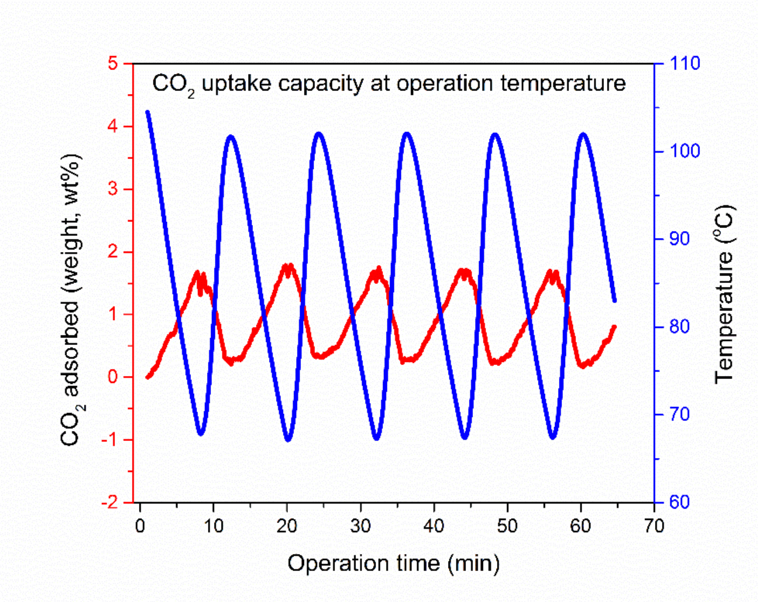

Supplementary Figure 12 The temperature-dependent adsorption ability of porous HCP-TiO_2-FG checked by TGA-DSC at CO_2 atmosphere.

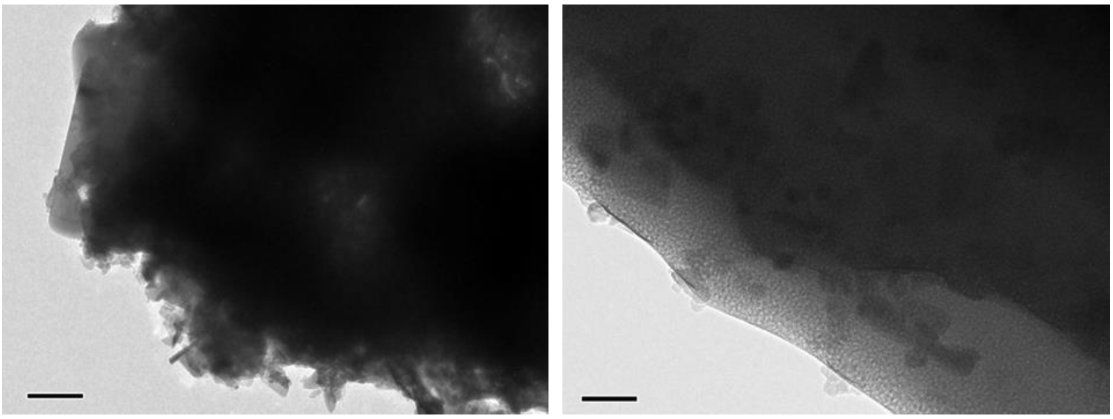

Supplementary Figure 13 TEM images of HCP-FG supported TiO_2 (TiO_2/HCP-FG) at different magnification. The scale bar are 200 nm in left image and 50 nm in right image.

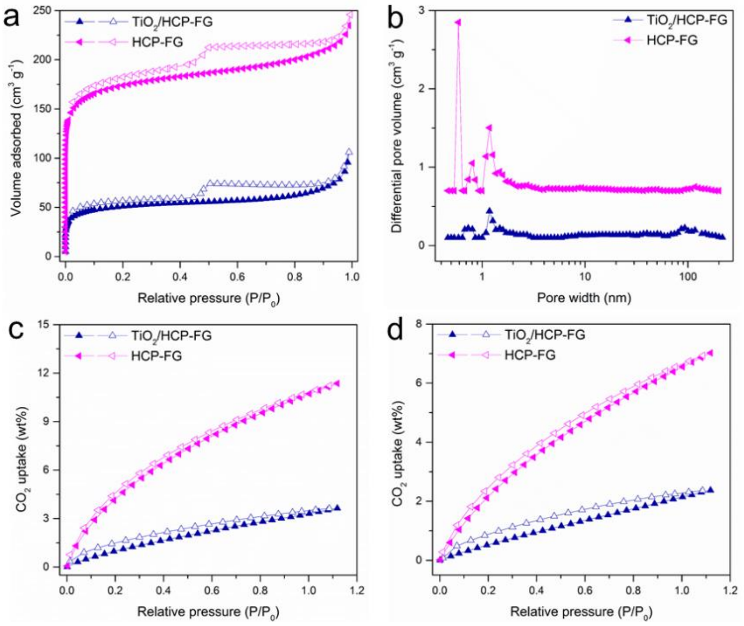

Supplementary Figure 14 The porosity and gas uptake of the contrastive samples, HCP-FG and TiO_2/HCP-FG. a Nitrogen adsorption and desorption isotherms at 77.3 K. **b** Pore size distribution that calculated using DFT methods (slit pore models, differential pore volumes). **c** Volumetric CO_2 adsorption isotherms and desorption isotherms up to 1.00 bar at 273.15 K. **d** Volumetric CO_2 adsorption isotherms and desorption isotherms up to 1.00 bar at 298.15 K.

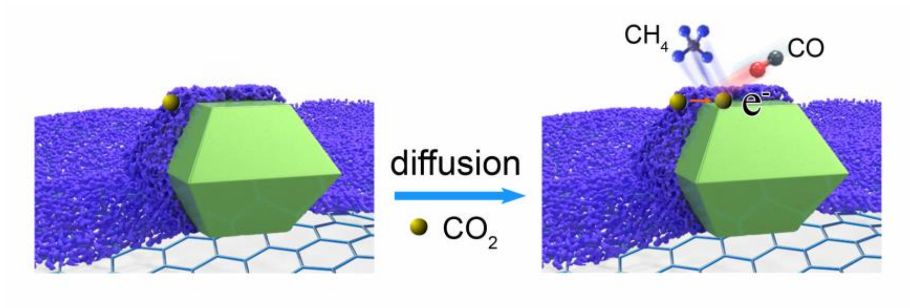

Supplementary Figure 15 Diagram of CO_2 diffusion from adsorptive sites to catalytic sites for conversion.

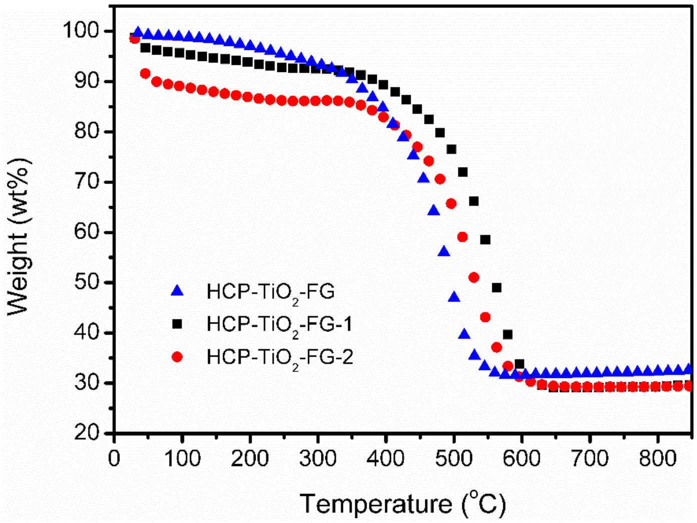

Supplementary Figure 16 Thermogravimetric analysis of HCP-TiO_2-FG, HCP-TiO_2-FG-1 and HCP-TiO_2-FG-2 at heating rate of 10 °C /min under air. The HCP-TiO_2-FG, HCP-TiO_2-FG-1 and HCP-TiO_2-FG-2 were synthesized by adding 20, 25 and 30 mg of *syn*-$PhPh_3$, respectively.

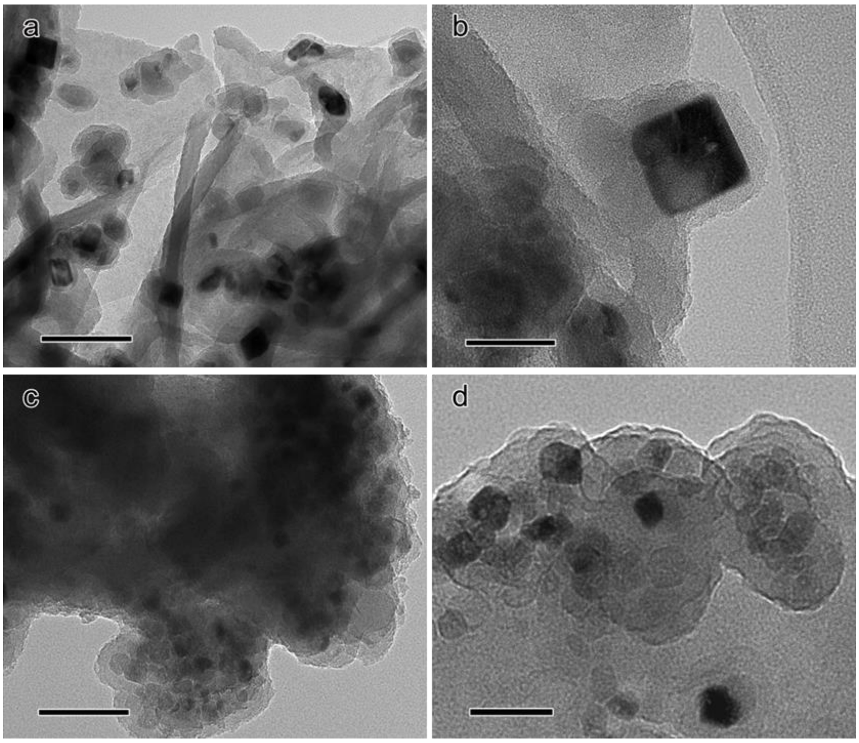

Supplementary Figure 17 TEM images of materials. TEM images of **a, b** HCP-TiO_2-FG-1 and **c, d** HCP-TiO_2-FG-2. The HCP-TiO_2-FG-1 and HCP-TiO_2-FG-2 were synthesized by adding 25 and 30 mg of *syn*-$PhPh_3$, respectively. The scale bar are 200 nm in **a**, 50 nm in **b**, 100 nm in **c**, and 50 nm in **d.**

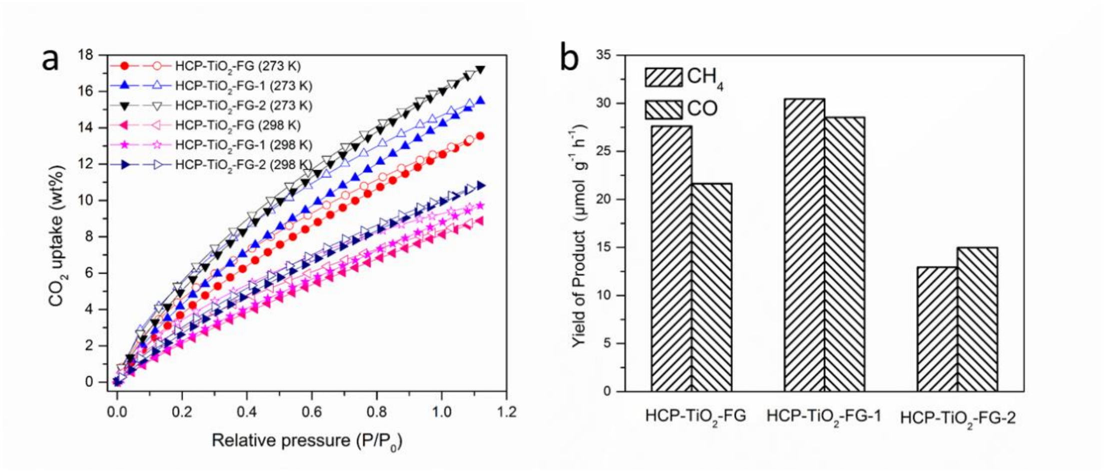

Supplementary Figure 18 a Effect of *syn*-PhPh$_3$ amount on the CO_2 uptake. **b** Effect of *syn*-PhPh$_3$ amount on the CH_4 and CO production rates over HCP-TiO_2-FG composite photocatalysts under visible-light (λ≥420 nm).

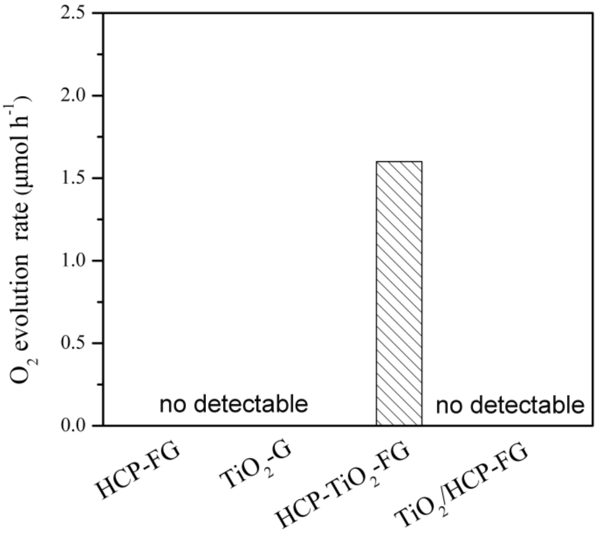

Supplementary Figure 19 The O_2 evolution rate during photocatalytic CO_2 conversion over photocatalysts under visible-light irradiation (λ≥420 nm). The quantitative measurement of O_2 gas was conducted using an optic fiber oxygen sensor (Ocean-Optics).

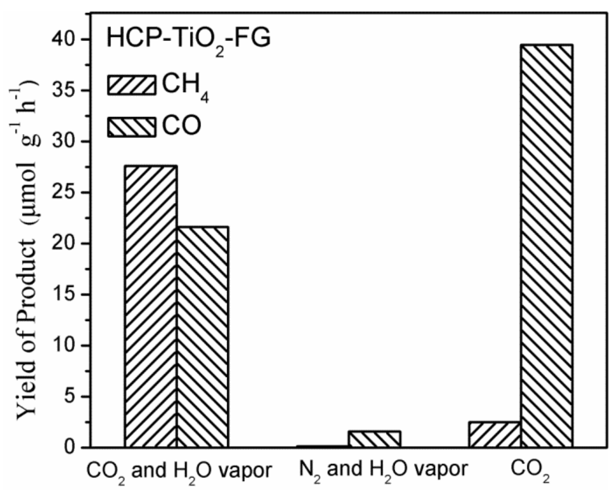

Supplementary Figure 20 The controlled experiments in photocatalytic CO_2 reduction.

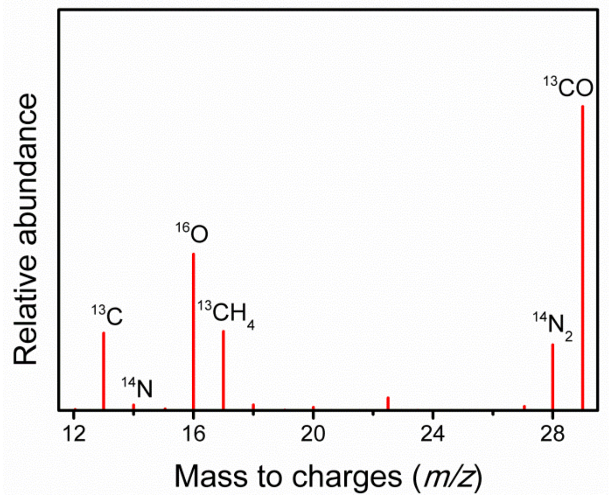

Supplementary Figure 21 GC-MS spectra of gas products after the photocatalytic reaction over HCP-TiO_2-FG under visible-light irradiation (λ≥420 nm). The isotopically labeled $^{13}CO_2$ was used as a substrate under normal experimental conditions.

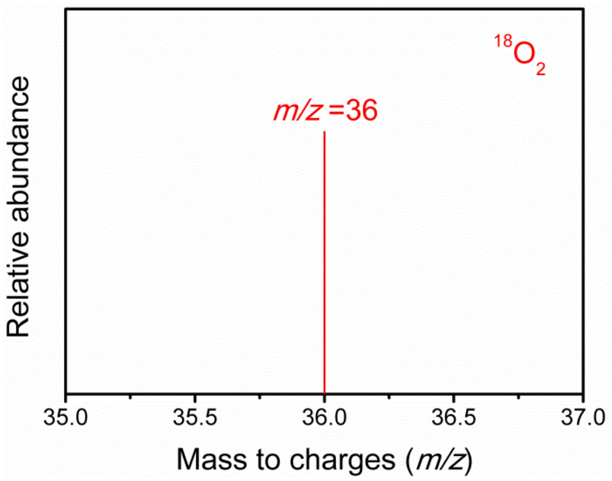

Supplementary Figure 22 GC-MS spectra of $^{18}O_2$ (m/z=36) after the photocatalytic reaction over HCP-TiO_2-FG under visible-light irradiation (λ≥420 nm). The isotopic labeled $H_2{}^{18}O$ was used as the substrate in normal experiment condition.

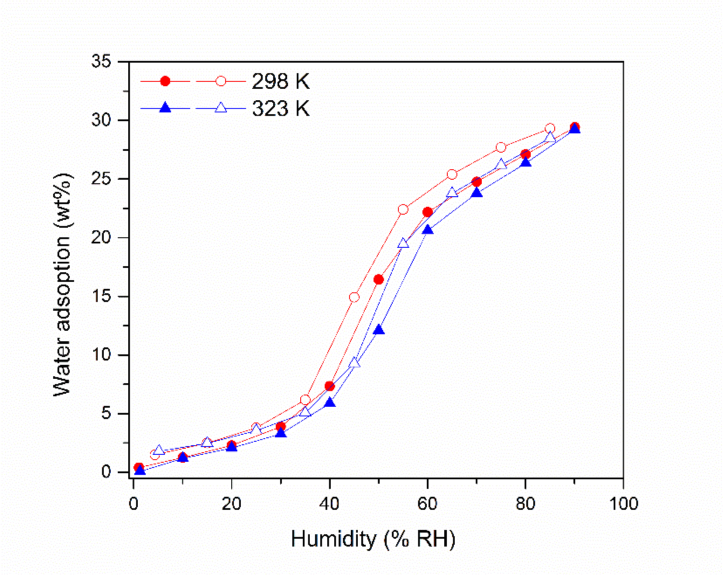

Supplementary Figure 23 The water adsorption of HCP-TiO_2-FG at different humidity.

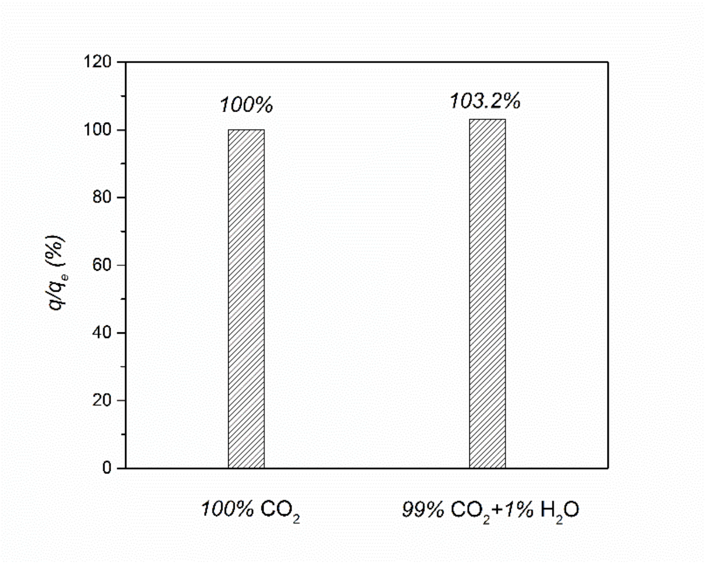

Supplementary Figure 24 CO_2 adsorption experiments, data in presence of water. q_e is the equilibrium adsorption capacity at pure CO_2 atmosphere with 1 bar. q/q_e represents fractional uptake at pure CO_2 atmosphere and mix atmosphere of *99%* CO_2+*1%* water vapors with 1 bar.

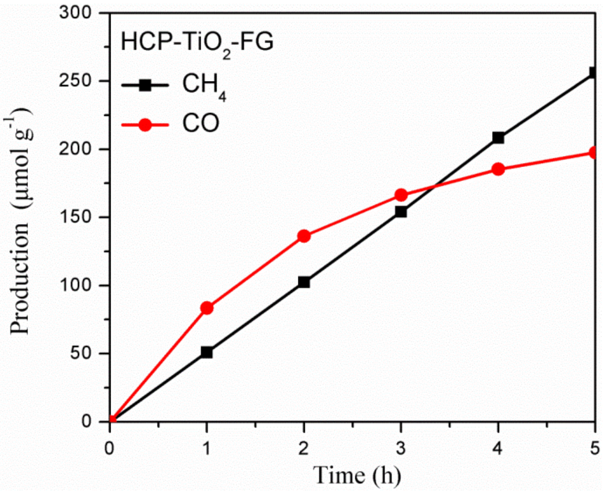

Supplementary Figure 25 Time-dependent production of CH_4 and CO in photocatalytic CO_2 reduction for HCP-TiO_2-FG under UV-light irradiation.

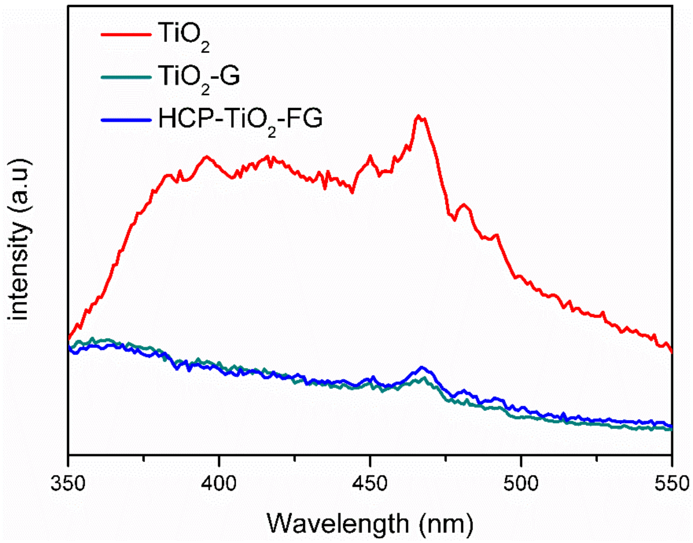

Supplementary Figure 26 Room temperature photoluminescence (PL) spectra of TiO_2, TiO_2-G, and HCP-TiO_2-FG.

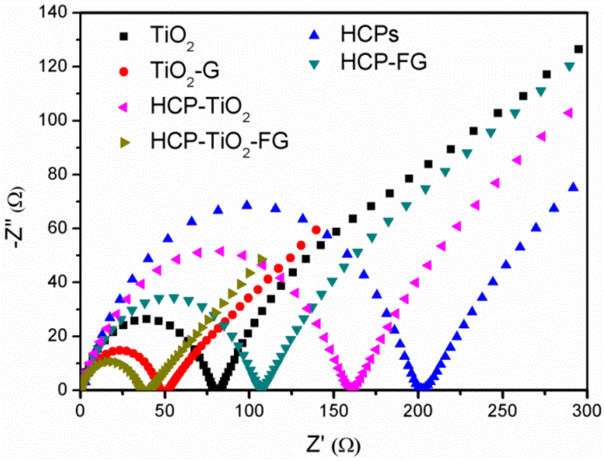

Supplementary Figure 27 Electrochemical impedance spectra (EIS) of the samples. Experimental condition: the samples dip-coated indium-tin oxide (ITO) glass as the work electrode, Ag/AgCl electrode as the reference electrode, a platinum wire as the counter electrode, 0.1 M KCl solution containing 5 mM $Fe(CN)_6^{3-}/Fe(CN)_6^{4-}$.

The semi-circle at high frequencies in the Nyquist diagrams is in accordance with that in the electron-transfer-limited process and the semi-circle diameter is equivalent to the electron-transfer resistance (R_{et}) across the electrode/electrolyte. The smaller R_{et} of TiO_2-FG than TiO_2 indicates that FG modification improves the electronic conductivity in the non-photoexcited state. It is reported that the low dimension materials such as graphene possesses very high electron mobility[3]. The smallest arc in HCP-TiO_2-FG composite structure suggests the most efficient charge transfer across the electrode/electrolyte interface among these materials.

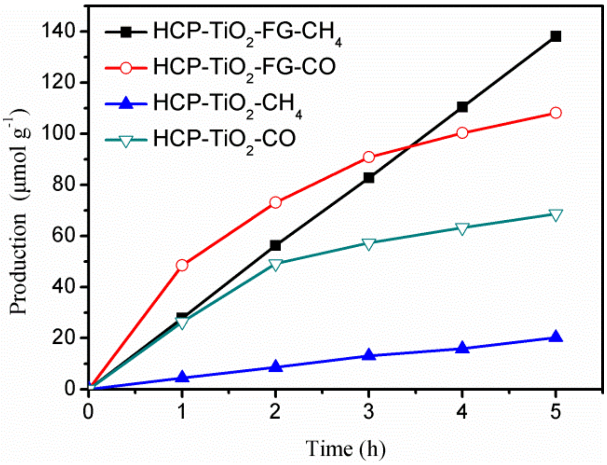

Supplementary Figure 28 Comparison of CH_4 and CO production rates between HCP-TiO_2 and HCP-TiO_2-FG photocatalysts under visible-light (λ≥420 nm).

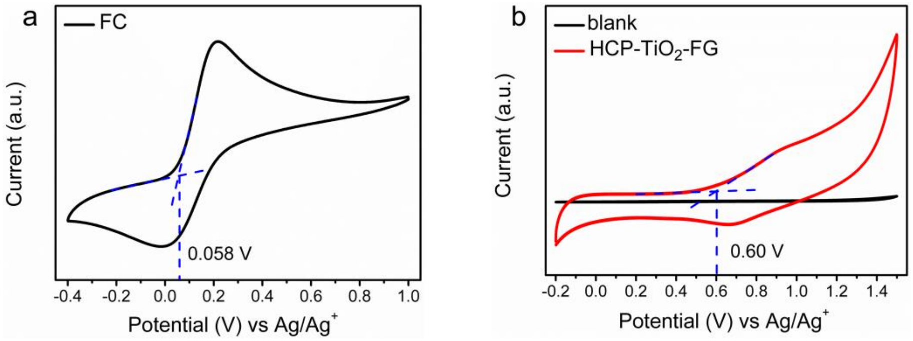

Supplementary Figure 29 Cyclic voltammetry (CV) measurements of ferrocence (FC) reference and HCP-TiO_2-FG catalyst on ITO glass electrode as the working electrode. The CV curves were recorded in N_2-saturated anhydrous acetonitrile containing 0.1 M tetrabutyl-ammonium hexafluorophosphate (Bu_4NPF_6). The ITO glass electrode with no catalyst coating was labeled as blank for comparison. Reference electrode: Ag/Ag^+ (0.01 M of $AgNO_3$ in acetonitrile). Counter electrode: Pt wire. Scan rate: 100 mV/s.

The energy gap of HCP-TiO_2-FG can be determined from the UV–vis spectrum in **Fig. 4d** using the equation $E_g = 1240/\lambda = 1240/530 = 2.34$ eV. The potentials levels *vs.* the vacuum level can be obtained from the above CV curves using the following equations:

Equations 2: HOMO = $-[E_{OX}-E(FC/FC^+)+4.8]$ eV= -(0.60-0.058+4.8) eV = -5.34 eV

Equations 3: LUMO = $-(HOMO+E_g)$ = -(-5.34+2.34) = -3.00 eV

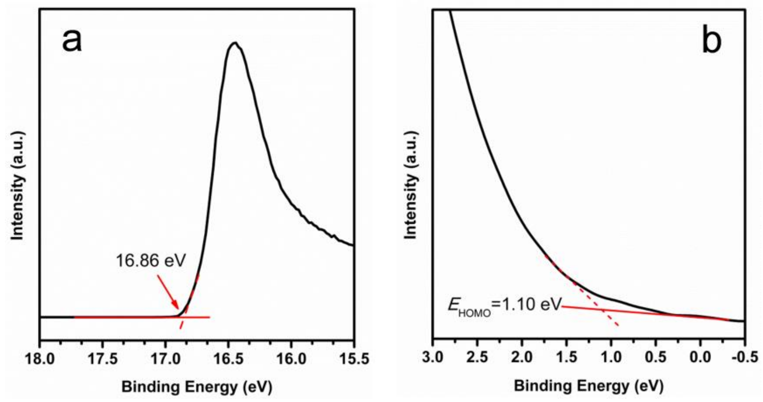

Supplementary Figure 30 Ultraviolet photoelectron spectroscopy (UPS) measurement. a secondary electron cutoff spectrum and **b** highest occupied molecular orbital (HOMO) energy level.

The work function (Φ) can be determined by the difference between the photon energy (21.2 eV) and the binding energy of the secondary cutoff edge.

Equations 4: Φ = 21.20-16.86 = 4.34 eV

The HOMO location is measured to be 1.10 eV below the Fermi level (E_F), corresponding to -5.44 eV vs vacuum level.

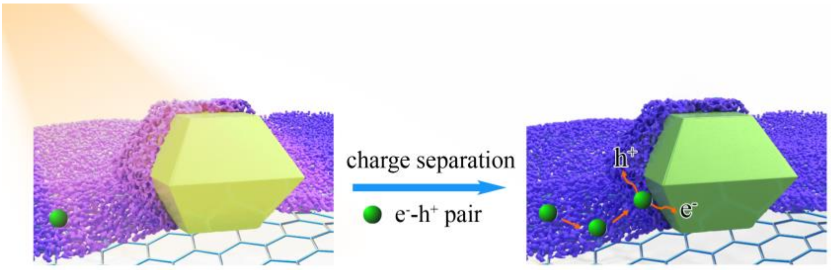

Supplementary Figure 31 Diagram of charge separation at the interface of HCP-FG with TiO_2.

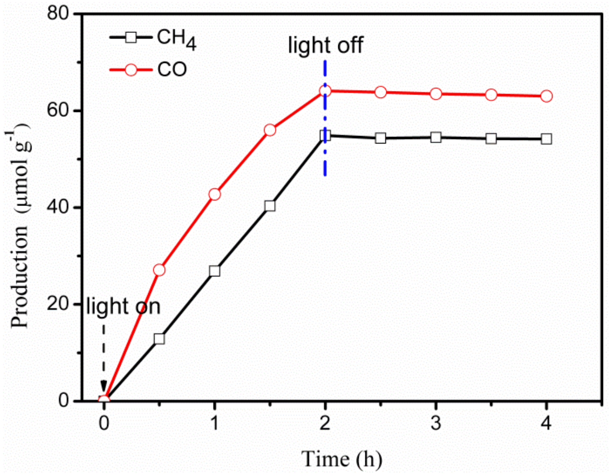

Supplementary Figure 32 Changes in CH_4 and CO production over the HCP-TiO_2-FG

photocatalyst under visible-light (λ≥420 nm) irradiation and dark conditions.

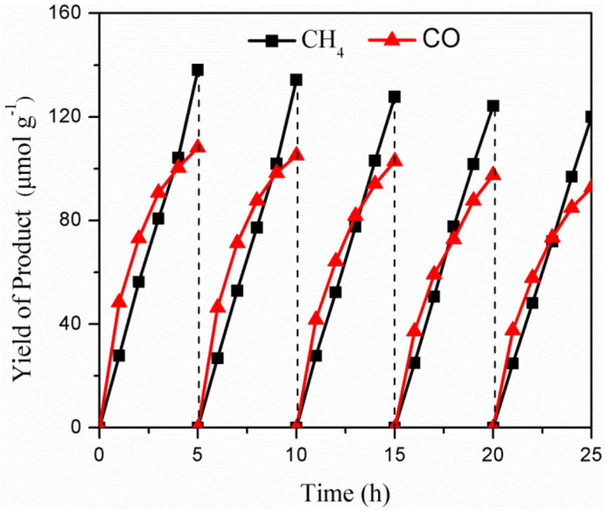

Supplementary Figure 33 CH_4 and CO evolution over HCP-TiO_2-FG composite within 5 cycles of constant photocatalytic reaction.

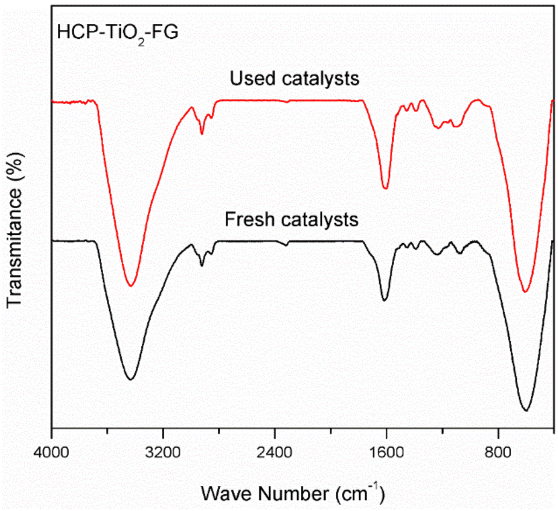

Supplementary Figure 34 FT-IR spectra of HCP-TiO_2-FG composite before and after photocatalytic CO_2 reduction.

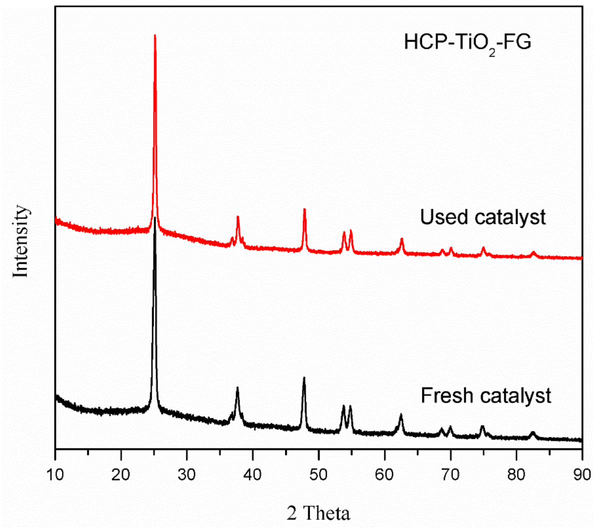

Supplementary Figure 35 XRD spectra of HCP-TiO_2-FG composite before and after photocatalytic CO_2 reduction.

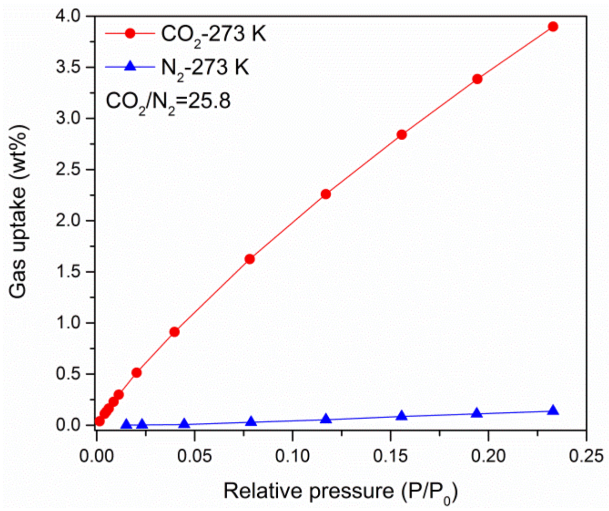

Supplementary Figure 36 CO_2 and N_2 adsorption isotherms of porous HCP-TiO_2-FG composite at 273 K. The HCP-TiO_2-FG photocatalyst exhibits a high CO_2/N_2 selectivity ratio of 25.8 calculated by the initial slopes of adsorption isotherms[4].

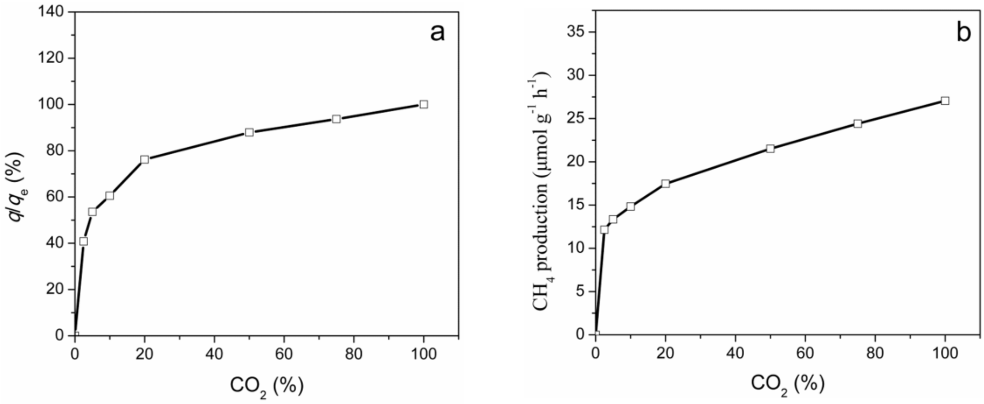

Supplementary Figure 37 Influence of the partial pressure of **a** CO_2 on the CO_2 uptake and **b** CH_4 production rate. The photocatalytic reactions were carried out in a batch system under standard atmospheric pressure. The partial pressure of CO_2 can be adjusted from 2.5% to 100% by varying the volume ratio of CO_2 to N_2. q_e is the equilibrium adsorption capacity at pure CO_2 atmosphere with 1 bar. q/q_e represents fractional uptake at different partial pressure of CO_2.

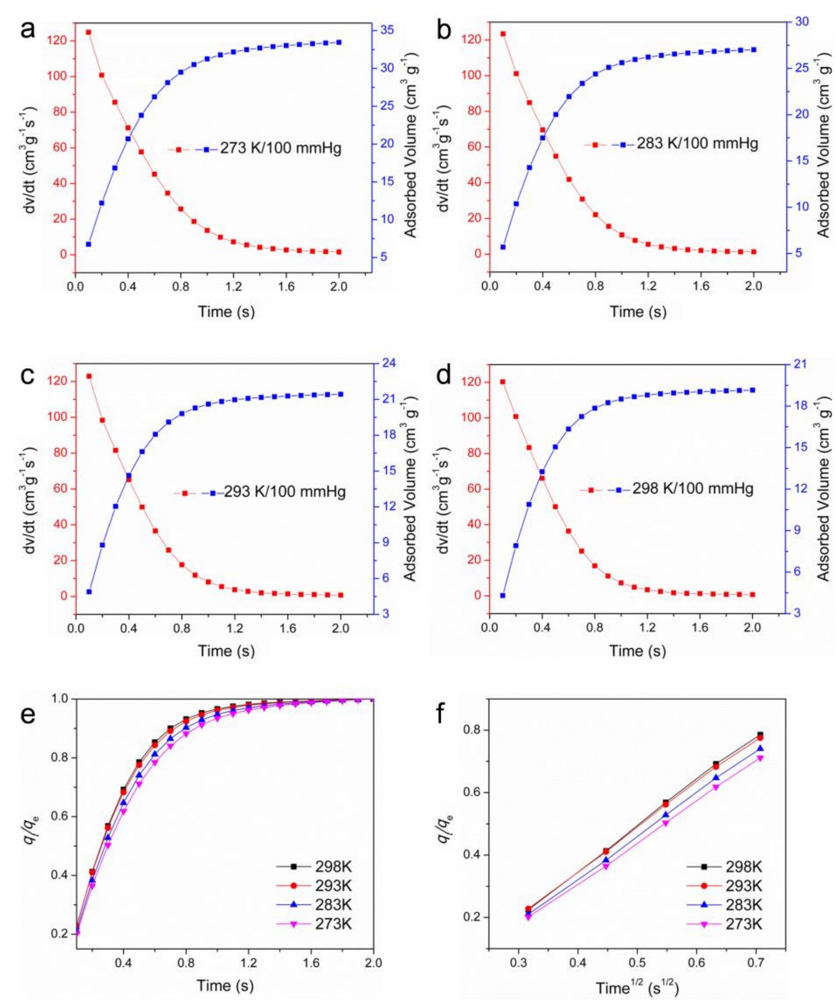

Supplementary Figure 38 a, b, c, d Adsorption kinetic curves of HCP-TiO_2-FG at different temperature. **e** Fractional adsorption uptake (q_t/q_e) at different temperature. **f** Plots of the fractional adsorption uptake (q_t/q_e) against the square root of adsorption time at different temperature.

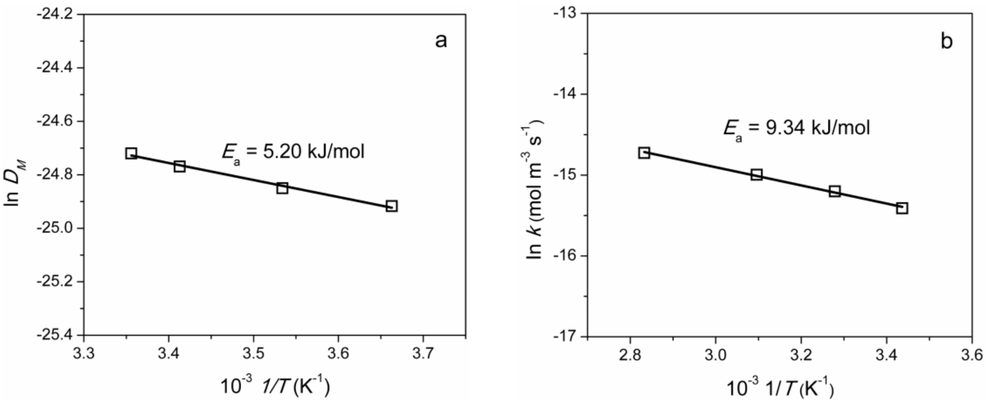

Supplementary Figure 39 Arrhenius plot of **a** CO_2 diffusivity and **b** CH_4 production rate over HCP-TiO_2-FG composite. The diffusion coefficient D_M is calculated using a microporous diffusion model:

Equations 5: $\frac{q_t}{q_e} \cong \frac{6}{r_c}\sqrt{\frac{D_M}{\pi}}\sqrt{t}$, r_c is the average particle size[5].

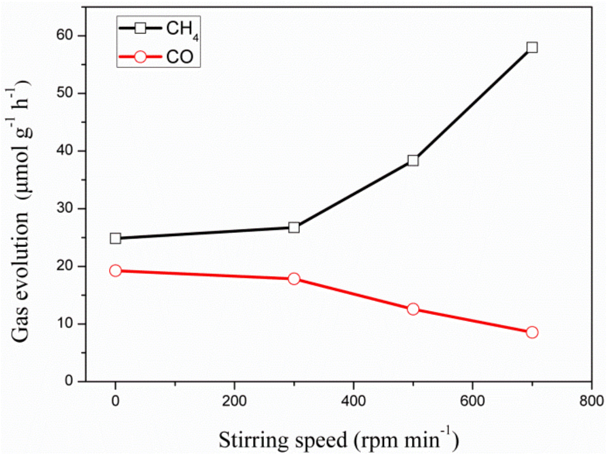

Supplementary Figure 40 Effect of stirring speed on CH_4 and CO production over the HCP-TiO_2-FG photocatalyst under visible-light (λ≥420 nm) irradiation.

Supplementary Table 1 Comparison of the activity of HCP-TiO_2-FG in the photocatalytic CO_2 reduction with the catalysts recently reported.

Photocatalyst	Light source	Products (μmol g^{-1} h^{-1})	Consumed electron number	Ref.
I. Gas-liquid-solid reaction system				
i. Traditional or porous photocatalyst with organic solvent				
$CsPbBr_3$ QD/GO, ethyl acetate	UV-vis[c]	CH_4 (2.5), CO (4.9), H_2 (0.13)	30	6
UiO-66/g-C_3N_4, MeCN/TEOA	λ > 400[a]	CO (9.9 μmol g_{CN}^{-1} h^{-1})	20	7
MOF-525-Co, MeCN/TEOA	λ > 400[a]	CO (200.6), CH_4 (36.67)	695	8
ii. Traditional or Porous photocatalyst without sacrificial reagent				
RGO-CoO_x/$BiVO_4$	λ > 420[a]	CO (~0.5)	1	9
$ZnGeO_4$/ZIF-8	UV-vis[h]	CH_3OH (0.22)	1	10
II. Gas-solid reaction system				
i. Photocatalyst with precious metal co-catalyst				
Au@TiO_2	UV-vis[a]	CH_4 (2.52), C_2H_6 (1.67)	20	11
Au-CNS-ZIF-9	UV-vis[a]	CO (7.66)	15	12
Pt-Cu_2O/TiO_2	UV[a]	CO (0.05), CH_4(1.42),	12	13
Rh/Cr_2O_3/GaN nanowires	UV-vis[a]	CH_4 (3.5), CO (4.8)	38	14
C_5H_5-RuH-O-TiO_2	λ > 420[a]	CH_4 (44 μL g^{-1} h^{-1})	16	15
Pd/*g*-C_3N_4	UV-vis[a]	CH_4 (0.4), CH_3OH (3.17)	22	16
Pd_7Cu_1-TiO_2	λ < 400[a]	CH_4 (19.6), CO (1.9), H_2 (1.45)	161	17
TiO_2-$PdH_{0.43}$ nanocubes	λ < 400[a]	CH_4(20.6), CO (9.5),O_2 (9.5), H_2 (40)	184	18
ii. Traditional photocatalyst without sacrificial reagent or precious metal co-catalyst				
TiO_2 ({001} and {101})	UV-vis[a]	CH_4(1.35)	11	19
$ZnIn_2S_4$	UV[a]	CO (33.2)	66	20
TiO_2/g-C_3N_4	λ > 400[a]	CH_4 (5.21), CO (0.84)	43	21
m-CeO_2/*g*-C_3N_4	UV-vis[a]	CH_4 (0.694), CO (0.508)	7	22

Carbon@TiO_2	UV-vis[a]	CH_4 (4.22), CH_3OH (9.11)	88	23
GO/oxygen-rich-TiO_2	λ > 400[h]	CH_4 (0.43), C_2H_6 (0.023), C_2H_4 (0.22), CO (1.13)	8	24
TiO_2/N-doped-RGO	λ > 400[b]	CO (44.56)	89	25
RGO-CdS	UV-vis[a]	CH_4 (2.51)	20	26
Amine-functionalized graphene/CdS	λ > 420[a]	CH_4 (2.84), CO (0.2)	23	27
RGO/g-C_3N_4	λ > 400[a]	CH_4 (0.579)	5	28
N-GQDs/V_o-NaTaON	λ > 400[a]	CH_4 (10), CO (43)	166	29
iii. Porous photocatalyst without sacrificial reagent or precious metal co-catalyst				
Mesoporous TiO_2	UV-vis[a]	CH_4 (0.192), CO (0.145)	2	30
Porous O-doped CN	λ > 420[d]	CH_3OH (0.88)	5	31
TiO_2/NH_2-UiO-66	λ > 325[g]	CO (4.25)	9	32
$Cu_3(BTC)_2$@TiO_2	λ < 400[a]	CH_4 (2.64 μmol g_{TiO2}^{-1} h^{-1})	21	33
CPO-27-Mg/TiO_2	365 nm[f]	CH_4 (2.35), CO (4.09)	27	34
Co-ZIF-9/TiO_2	UV-vis[a]	CH_4 (1.98), CO (17.58), H_2 (2.6)	51	35
g-C_3N_4/ZIF-8	UV-vis[a]	CH_3OH (0.75)	5	36
HCP-TiO_2-FG	λ > 420[a]	CH_4 (27.62), CO (21.63)	264	This work
HCP-TiO_2-FG	UV-vis[a]	CH_4 (51.23), CO (39.51)	489	This work

[a] 300 W Xe lamp. [b] 400 W Xe lamp. [c] 100 W Xe lamp. [d] 350 W Xe lamp. [e] Hg-Xe arc lamp 200 W. [f] 4W UV lamp [g]150 W Xe lamp. [h] 500 W Xe lamp. The rate of consumed electron number for the reduced products of CO_2 ($R_{electron}$) = $2R(CO)+6R(CH_3OH)+8R(CH_4)+\ldots$, where R(CO), $R(CH_3OH)$, $R(CH_4)$ are the formation rates (μmol g^{-1} h^{-1}) of the CO, CH_3OH, CH_4, respectively.

To explain the effect of CO_2 uptake, several porous photocatalysts with relatively high CO_2 conversion were used as controls. $Cu_3(BTC)_2$@TiO_2 with core-shell structure exhibited a CH_4 production rate of 2.64 μmol $g(TiO_2)^{-1}$ h^{-1} under UV-light illumination, which was more than 5 times of bare TiO_2[33]. The MOFs material with highest CO_2 uptake, CPO-27-Mg, was found to enhance the CO and CH_4 yields of TiO_2 under UV-light from 2.25 and 1.37 μmol g^{-1} h^{-1} to 4.09 and 2.35 μmol g^{-1} h^{-1}, respectively[34]. The CO_2 reduction over Co-ZIF-9/TiO_2 composites included CO, CH_4, and H_2 evolution with a rate of 17.58, 1.98, and 2.6 μmol g^{-1} h^{-1} under UV-visible light, which was

about 2.1 times higher than that of pure TiO_2[35].

Converting CO_2 into CH_4 is relatively difficult since the reactivity of the adsorbed CO_2 molecules should be good enough to accept eight electrons and eight protons to break the C-O bonds and form the C-H bonds[37]. To the best of our knowledge, the highest CH_4 production rates over photocatalysts were achieved with the assistance of precious metal co-catalysts, e.g. 19.6 μmol g^{-1} h^{-1} over Pd_7Cu_1-loaded TiO_2 and 20.6 μmol g^{-1} h^{-1} over TiO_2-$PdH_{0.43}$ under UV-light[17, 18]. The high CH_4 production could be ascribed to the improved reactivity of CO_2 molecules on the surface of precious metal.

Supplementary Table 2 The porosity and gas uptake of the samples.

Sample	S_{BET}[a] (m^2 g^{-1})	S_L[b] (m^2 g^{-1})	PV[c] (cm^3 g^{-1})	MPV[d] (cm^3 g^{-1})	CO_2 uptake[e] (wt %)	CO_2 uptake[f] (wt %)
TiO_2	114	156	0.157	0.006	2.85	1.91
TiO_2-G	136	192	0.180	0.009	2.87	1.89
HCP-FG	593	786	0.380	0.195	10.90	6.71
TiO_2/HCP-FG	178	236	0.164	0.046	3.31	2.14
HCP-TiO_2-FG	988	1312	0.693	0.306	12.87	8.64

[a] Surface area calculated from nitrogen adsorption isotherms at 77.3 K using BET equation. [b] Surface area calculated from nitrogen adsorption at 77.3 K using Langmuir equation. [c] Pore volume calculated from nitrogen isotherm at P/P_0=0.995, 77.3 K. [d] Micropore volume calculated from the nitrogen isotherm at P/P_0=0.050. [e] CO_2 uptake determined volumetrically using a Micromeritics ASAP 2020 M analyzer at 1.00 bar and 273.15 K. [f] CO_2 uptake determined volumetrically using a Micromeritics ASAP 2020 M analyzer at 1.00 bar and 298.15 K.

Supplementary Table 3 Comparison of the surface area and CO_2 uptake ability of the reported photocatalysts for CO_2 reduction.

Photocatalyst	S_{BET} (m^2 g^{-1})	S_L (m^2 g^{-1})	CO_2 uptake	**Ref.**
I. Semiconductor-graphene composite				
TiO_2/N-doped-RGO	59	---	6.3% (195K)	25
RGO-CdS	51	---	0.57% (298K)	26
RGO/g-C_3N_4	92.1	---	---	28
AgBr/CN/N-doped-G	81.6	---	---	38
Graphene-TiO_2	97.6	---	1.8% (298K)	39
II. Porous photocatalyst				
UiO-66/g-C_3N_4	---	1315	6.42% (298K)	7

$ZnGeO_4$/ZIF-8	319	---	---	10
Mesoporous TiO_2	212	---	---	30
Porous O-doped CN	36	---	0.19% (298K)	31
TiO_2/NH_2-UiO-66	202	---	1.58% (298K)	32
$Cu_3(BTC)_2$@TiO_2	756	---	9.6% (298K)	33
CPO-27-Mg/TiO_2	416.8	-----	20.8% (273K)	34
g-C_3N_4/ZIF-8	19	---	0.17% (298K)	36
HCP-TiO_2-FG	988	1312	8.64% (298K)	This work

Supplementary Table 4 The porous property and CO_2 conversion efficiency of the HCP-TiO_2-FG composites with different amount of *syn*-$PhPh_3$.

Sample	S_{BET}[a] ($m^2\ g^{-1}$)	CO_2 uptake[b] (wt %)	PV[c] ($cm^3\ g^{-1}$)	Visible-light irradiation (μmol $g^{-1}\ h^{-1}$)		
				$r(CH_4)$[d]	r(CO)[e]	R_e[f]
HCP-TiO_2-FG	988	12.87	0.693	27.62	21.63	264
HCP-TiO_2-FG-1	1136	14.65	0.758	30.45	28.52	301
HCP-TiO_2-FG-2	1362	16.45	0.799	12.94	14.97	133

Supplementary Methods

Synthesis of graphene oxide (GO) *via* a modified hummer's method. Under an ice-water bath, 2 g graphite and 1 g $NaNO_3$ were successively added to 75 mL of conc. H_2SO_4. After stirring for 10 min, 10 g $KMnO_4$ was added slowly in portions to maintain the reaction temperature blow 10 °C. The mixture was heated up to 40 °C and stirred for 45 min, and then 75 mL distilled water was added slowly. The reaction temperature was raised to 95 °C and maintained for 15 min, and the color of suspension changed from brown to uniform golden-yellow. The mixture was cooled followed by the addition of 150 mL distilled water and 15 mL H_2O_2. The GO was obtained by filtration and washing with 300 mL 1% HCl and abundant water. The GO was dispersed in 200 mL distilled water and dialyzed against water until the dialysate became neutral. Finally, the GO dispersion was diluted to 5 L and sonicated for 30 min, and the GO concentrate about 8 mg/mL was obtained by concentrating the supernatant of GO diluent.

Synthesis of lamellar protonated titanate (LPT) as TiO_2 precursor.

9 mL of tetrabutyl titanate was dissolved in 66 mL of absolute ethanol, and then the solution was added dropwise into 90 mL of deionized water under magnetic stirring at room temperature. After complete addition, the white suspension was mechanically agitated at 70 °C for about 2 h to obtain

a condensed suspension. 300 mL of 1 M NaOH was then added into the above suspension, and the mixture was further stirred for 12 h under air-tight conditions. After centrifugation, deionized water and isopropanol were used in turn to thoroughly wash the white precipitate. Thus the wet LPT precursor was obtained for future use.

Supplementary References

1. Zhang, D., Li, G., Yang, X. & Yu, J. C. A micrometer-size TiO_2 single-crystal photocatalyst with remarkable 80% level of reactive facets. *Chem. Commun.* **29**, 4381-4383 (2009).
2. Yuan, K. *et al.* Straightforward generation of pillared, microporous graphene frameworks for use in supercapacitors. *Adv. Mater.* **27**, 6714-6721 (2015).
3. Novoselov, K. S. *et al.* Electric field effect in atomically thin carbon films. *Science* **306**, 666-669 (2004).
4. Luo, Y., Li, B., Wang, W., Wu, K. & Tan, B. Hypercrosslinked aromatic heterocyclic microporous polymers: a new class of highly selective CO_2 capturing materials. *Adv. Mater.* **24**, 5703-5707 (2012).
5. Zhao, Z., Li, Z. & Lin, Y. S. Adsorption and diffusion of carbon dioxide on metal-organic framework (MOF-5). *Ind. Eng. Chem. Res.* **48**, 10015-10020 (2009).
6. Xu, Y. F. *et al.* A $CsPbBr_3$ perovskite quantum dot/graphene oxide composite for photocatalytic CO_2 reduction. *J. Am. Chem. Soc.* **139**, 5660-5663 (2017).
7. Shi, L., Wang, T., Zhang, H., Chang, K. & Ye, J. Electrostatic self-assembly of nanosized carbon nitride nanosheet onto a zirconium metal-organic framework for enhanced photocatalytic CO_2 reduction. *Adv. Funct. Mater.* **25**, 5360-5367 (2015).
8. Zhang, H. *et al.* Efficient visible-light-driven carbon dioxide reduction by a single-atom implanted metal-organic framework. *Angew. Chem. Int. Ed.* **55**, 14308-14312 (2016).
9. Iwase, A., Yoshino, S., Takayama, T., Ng, Y. H., Amal, R. & Kudo, A. Water splitting and CO_2 reduction under visible light irradiation using Z-scheme systems consisting of metal sulfides, CoO_x-loaded $BiVO_4$, and a reduced graphene oxide electron mediator. *J. Am. Chem. Soc.* **138**, 10260-10264 (2016).
10. Liu, Q. *et al.* ZIF-8/Zn_2GeO_4 nanorods with an enhanced CO_2 adsorption property in an aqueous medium for photocatalytic synthesis of liquid fuel. *J, Mater. Chem. A* **1**,11563-11569 (2013).
11. Tu, W., Zhou, Y., Li, H., Li, P. & Zou, Z. Au@TiO_2 yolk-shell hollow spheres for plasmon-induced photocatalytic reduction of CO_2 to solar fuel via a local electromagnetic field. *Nanoscale* **7**, 14232-14236 (2015).
12. Zhou, H. *et al.* Biomimetic polymeric semiconductor based hybrid nanosystems for artificial photosynthesis towards solar fuels generation via CO_2 reduction. *Nano Energy* **25**, 128-135 (2016).
13. Xiong, Z. *et al.* Selective photocatalytic reduction of CO_2 into CH_4 over Pt-Cu_2O TiO_2 nanocrystals: The interaction between Pt and Cu_2O cocatalysts. *Appl. Catal. B-Environ.* **202**, 695-703 (2017).

14. AlOtaibi, B., Fan, S., Wang, D., Ye, J. & Mi, Z. Wafer-level artificial photosynthesis for CO_2 reduction into CH_4 and CO Using GaN nanowires. *ACS Catal.* **5**, 5342-5348 (2015).
15. Huang H, Lin J, Zhu G, Weng Y, Wang X, Fu X*, et al.* A long-lived mononuclear cyclopentadienyl ruthenium complex grafted onto anatase TiO_2 for efficient CO_2 photoreduction. *Angew. Chem. Int. Ed.* **55**, 8314-8318 (2016).
16. Cao, S. W, Li, Y., Zhu, B. C., Jaroniec M. & Yu, J. G. Facet effect of Pd cocatalyst on photocatalytic CO_2 reduction over g-C_3N_4. *J. Catal.* **349**, 208-217 (2017).
17. Long, R. *et al.* Isolation of Cu atoms in Pd lattice: forming highly selective sites for photocatalytic conversion of CO_2 to CH_4. *J. Am. Chem. Soc.* **139**, 4486-4492 (2017).
18. Zhu, Y. Z. *et al.* Hydriding Pd cocatalysts: an approach to giant enhancement on photocatalytic CO_2 reduction into CH_4. *Nano Res.* **10**, 3396-3406 (2017).
19. Yu, J., Low, J., Xiao, W., Zhou, P. & Jaroniec, M. Enhanced photocatalytic CO_2-reduction activity of anatase TiO_2 by coexposed {001} and {101} facets. *J. Am. Chem. Soc.* **136**, 8839-8842 (2014).
20. Jiao X. *et al.* Defect-mediated electron-hole separation in one-unit-cell $ZnIn_2S_4$ Layers for boosted solar-driven CO_2 reduction. *J. Am. Chem. Soc.* **139**, 7586-7594 (2017).
21. Li, K., Peng, B., Jin, J., Zan, L. & Peng, T. Carbon nitride nanodots decorated brookite TiO_2 quasi nanocubes for enhanced activity and selectivity of visible-light-driven CO_2 reduction. *Appl. Catal. B: Environ.* **203**, 910-916 (2017).
22. Li, M. *et al.* Mesostructured CeO_2/g-C_3N_4 nanocomposites: remarkably enhanced photocatalytic activity for CO_2 reduction by mutual component activations. *Nano Energy* **19**, 145-155 (2016).
23. Wang, W. K., Xu, D. F., Cheng, B., Yu, J. G. & Jiang, C. J. Hybrid carbon@TiO_2 hollow spheres with enhanced photocatalytic CO_2 reduction activity. *J. Mater. Chem. A* **5**, 5020-5029 (2017).
24. Tan, L. L., Ong, W. J., Chai, S. P. & Mohamed, A. R. Photocatalytic reduction of CO_2 with H_2O over graphene oxide supported oxygen-rich TiO_2 hybrid photocatalyst under visible light irradiation: Process and kinetic studies. *Chem. Eng. J.* **308**, 248-255 (2017).
25. Lin, L. Y., Nie, Y., Kavadiya, S., Soundappan, T. & Biswas, P. N-doped reduced graphene oxide promoted nano TiO_2 as a bifunctional adsorbent/photocatalyst for CO_2 photoreduction: Effect of N species. *Chem. Eng. J.* **316,** 449-460 (2017).
26. Yu, J., Jin, J., Cheng, B. & Jaroniec, M. A noble metal-free reduced graphene oxide-CdS nanorod composite for the enhanced visible-light photocatalytic reduction of CO_2 to solar fuel. *J.Mater. Chem. A* **2**, 3407-3416 (2014).
27. Cho, K. *et al.* Amine-functionalized graphene/CdS composite for photocatalytic reduction of CO_2. *ACS Catal.* **7**, 7064-7069 (2017).
28. Yu, K. *et al.* Preparation of an ultrathin 2D/2D rGO/g-C_3N_4 nanocomposite with enhanced visible-light-driven photocatalytic performance. *RSC Adv.* **7**, 36793-36799 (2017).
29. Hou, J. G. *et al.* Perovskite-based nanocubes with simultaneously improved visible-light absorption and charge separation enabling efficient photocatalytic CO_2 reduction. *Nano Energy* **30**, 59-68 (2016).

30. Wang, T. *et al.* Photoreduction of CO_2 over the well-crystallized ordered mesoporous TiO_2 with the confined space effect. *Nano Energy* **9**, 50-60 (2014).
31. Fu, J. *et al.* Hierarchical porous O-doped g-C_3N_4 with enhanced photocatalytic CO_2 reduction activity. *Small* **13**, 1603938 (2017).
32. Crake, A., Christoforidis, K. C., Kafizas, A., Zafeiratos S. & Petit, C. CO_2 capture and photocatalytic reduction using bifunctional TiO_2/MOF nanocomposites under UV–vis irradiation. *Appl. Catal. B: Environ.* **210**, 131-140 (2017).
33. Li, R. *et al.* Integration of an inorganic semiconductor with a metal-organic framework: a platform for enhanced gaseous photocatalytic reactions. *Adv. Mater.* **26**, 4783-4788 (2014).
34. Wang, M., Wang, D. & Li, Z. Self-assembly of CPO-27-Mg/TiO_2 nanocomposite with enhanced performance for photocatalytic CO_2 reduction. *Appl. Catal. B-Environ.* **183**, 47-52 (2016).
35. Yan, S. *et al.* Co-ZIF-9/TiO_2 nanostructure for superior CO_2 photoreduction activity. *J. Mater. Chem A* **4**, 15126-15133 (2016).
36. Liu, S., Chen, F., Li, S., Peng, X. & Xiong, Y. Enhanced photocatalytic conversion of greenhouse gas CO_2 into solar fuels over g-C_3N_4 nanotubes with decorated transparent ZIF-8 nanoclusters. *Appl. Catal. B: Environ.* **211**, 1-10 (2017).
37. Ji, Y. & Luo, Y. Theoretical study on the mechanism of photoreduction of CO_2 to CH_4 on the anatase TiO_2(101) surface. *ACS Catal.* **6**, 2018-2025 (2016).
38. Li, H. Y., Gan, S. Y., Wang, H. Y., Han, D. X. & Niu, L. Intercorrelated superhybrid of AgBr supported on graphitic-C_3N_4-decorated nitrogen-doped graphene: high engineering photocatalytic activities for water purification and CO_2 reduction. *Adv. Mater.* **27**, 6906-6913 (2015).
39. Tu, W. *et al.* An in situ simultaneous reduction-hydrolysis technique for fabrication of TiO_2-graphene 2D sandwich-like hybrid nanosheets: graphene-promoted selectivity of photocatalytic-driven hydrogenation and coupling of CO_2 into methane and ethane. *Adv. Funct. Mater.* **23**, 1743-1749 (2013).
