# Supplementary material for: Porous hypercrosslinked polymer-TiO2-graphene composite photocatalysts for visible-light-driven CO2 conversion
Source: Nat Commun. 2019 Feb 8;10:676. doi: 10.1038/s41467-019-08651-x (PMC6368626; doi:10.1038/s41467-019-08651-x)
Supplement: Supplementary file 3 — Description of Additional Supplementary Files [file 41467_2019_8651_MOESM3_ESM.pdf]

Description of Additional Supplementary Files

File Name: Supplementary Movie 1
Description: Three dimensional TEM movie of the HCP-TiO_2-FG composite by rotating the angle of sample holder also are provided.
